# Signal Fluctuations and the Information Transmission Rates in Binary Communication Channels

**DOI:** 10.3390/e23010092

**Published:** 2021-01-10

**Authors:** Agnieszka Pregowska

**Affiliations:** Institute of Fundamental Technological Research, Polish Academy of Sciences, Pawinskiego 5B, 02-106 Warsaw, Poland; aprego@ippt.pan.pl; Tel.: +48-22-826-12-81 (ext. 417)

**Keywords:** information source, information transmission rate, fluctuations, Shannon entropy, spike-trains, standard deviation

## Abstract

In the nervous system, information is conveyed by sequence of action potentials, called spikes-trains. As MacKay and McCulloch suggested, spike-trains can be represented as bits sequences coming from Information Sources (IS). Previously, we studied relations between spikes’ Information Transmission Rates (ITR) and their correlations, and frequencies. Now, I concentrate on the problem of how spikes fluctuations affect ITR. The IS are typically modeled as stationary stochastic processes, which I consider here as two-state Markov processes. As a spike-trains’ fluctuation measure, I assume the standard deviation σ, which measures the average fluctuation of spikes around the average spike frequency. I found that the character of ITR and signal fluctuations relation strongly depends on the parameter s being a sum of transitions probabilities from a no spike state to spike state. The estimate of the Information Transmission Rate was found by expressions depending on the values of signal fluctuations and parameter *s*. It turned out that for smaller s<1, the quotient $\frac{ITR}{\sigma }$ has a maximum and can tend to zero depending on transition probabilities, while for 1<s, the $\frac{ITR}{\sigma }$ is separated from 0. Additionally, it was also shown that ITR quotient by variance behaves in a completely different way. Similar behavior was observed when classical Shannon entropy terms in the Markov entropy formula are replaced by their approximation with polynomials. My results suggest that in a noisier environment (1<s), to get appropriate reliability and efficiency of transmission, IS with higher tendency of transition from the no spike to spike state should be applied. Such selection of appropriate parameters plays an important role in designing learning mechanisms to obtain networks with higher performance.

## 1. Introduction

Information transmission processes in natural environments are typically affected by signal fluctuations due to the presence of noise-generating factors [1]. It is particularly visible in biological systems, in particular during signal processing in the brain [2,3,4,5,6]. The physical information carriers in the brain are small electrical currents [7]. Specifically, the information is carried by sequences of action potentials also called spikes-trains. Assuming some time resolution, MacKay and McCulloch proposed a natural encoding method that associates to each spike-train a binary sequence [8]. Thus, the information is represented by a sequence of bits which, from a mathematical point of view, can be treated as a trajectory of some stochastic process [9,10].

In 1948, C. Shannon developed his famous Communication Theory where he introduced the concept of information and its quantitative measure [11]. The occurrences of both inputs transmitted through a communications channel and output symbols are described by sequences of random variables that define already stochastic processes and form some Information Sources [9,12]. Following this line, to characterize the amount of information transmitted per symbol the Information Transmission Rate (ITR) is applied.

Spike-trains Information Sources are often modeled as Poisson point processes [13,14]. Poisson point processes provide a good approximation of the experimental data, especially when the refractory time scale or, more generally, any memory time scale in the spike generation mechanism is short compared to the time scales such as mean interspike interval. The use of Poisson processes to model spike trains has been proposed from the earliest descriptions [15,16] due to the proportional relationship between the mean and variance of multiple neuronal responses. The Poisson property has been observed for many experimental data [17]. On the other hand, it is known that such processes exhibit Markov properties [18,19]. This is because in these processes when describing spikes arrival times, current time, and the time from the last spike are primarily taken into account [20]. There is a number of papers devoted to the modeling of spike-trains by different types of Markov processes (Markov Interval Models, Hidden Markov processes, Poisson Point processes) successfully applied to a variety of experimental data [20,21,22].

Description of complex systems dynamics, from financial markets [23] to the neural networks of living beings [24,25], requires appropriate mathematical tools. Among them, there are stochastic processes, Information Theory and statistical methods and recently, fuzzy numbers [26,27]. In recent years, to limit or even to exploit the effect of noise and fluctuations on information transmission efficiency extensive effort has been conducted, specifically to design spiking neuronal networks (SNNs) with appropriate learning mechanisms [28,29,30,31,32]. Moreover, different models of a neuron have been used to address noise resistance [33]. Traditionally, the complex nature of systems is characterized, mostly due to the presence of noise, by using fluctuations, variations, or other statistical tools [18,19,34,35,36]. The natural measure of fluctuations should, in general, reflect oscillations around the mean average value of the signal. Therefore, in most systems in physics, economics, fluid mechanics, fluctuations are most often quantifyied using the Standard Deviation [37,38,39].

In this paper, I analyze the relationship between the Information Transmission Rate of signals coming from a time-discrete two-states Markov Information Source and these signal fluctuations. As a spike-trains’ fluctuation measure, I consider the Standard Deviation of encoded spikes. Moreover, to gain a better insight, I have also analyzed the case when the ITR is referred to the signals Variance *V* instead of the Standard Deviation σ.

In the analysis of neuronal coding, specifically when studying neuronal signals, finding relationships between these signals’ main characteristics is of great importance [10]. Addressing this issue in our previous papers, I successively analyze the relations:

(1) between signal Information Transmission Rates (also Mutual Information) and signal correlations [40]. I show that neural binary coding cannot be captured by straightforward correlations among input and output signals.
(2) between signals information transmission rates and signal firing rates (spikes’ frequencies) [41]. By examining this dependence, I have found the conditions in which temporal coding rather than rate coding is used. It turned out that this possibility depends on the parameter characterizing the transition from state to state.
(3) between information transmission rates of signals (which are (auto)correlated) coming from Markov information sources and information transmission rates of signals coming from corresponding (to this Markov processes) Bernoulli processes. Here, “corresponding” means limiting the Bernoulli process with stationary distributions of these Markov processes [42]. I have shown in the case of correlated signals that the loss of information is relatively small, and thus temporal codes, which are more energetically efficient, can replace rate codes effectively. These results were confirmed by experiments.

In this paper, I consider the next important issue, namely I study the relation between Information Transmission Rates of signals and fluctuations of these signals [40]. I found that also the character of the relation between ITR and signal fluctuations strongly depends on the parameter *s*. It turned out that for small *s*(s<1), the quotient $\frac{ITR}{\sigma }$ has a maximum and tends to zero when the probability of transition from no spike state to spike state never reaches 0. While for large enough *s*, the quotient $\frac{ITR}{\sigma }$ is limited from below. A similar result appears when the Shannon entropy formula is replaced by appropriate polynomials.

On the other hand, I found that when I refer the quotient $\frac{ITR}{\sigma }$ to σ, i.e., when I consider, in fact, the quotient $\frac{ITR}{V}$, this quotient behaves in a completely different way. This behavior is not regular. Specifically, I observed that for 1<s, there is some range of parameter *s* for which $\frac{ITR}{V}$ has a few local extremas, in opposition to the case $\frac{ITR}{\sigma }$.

The paper is organized as follows. In Section 2, I briefly recall Shannon Information Theory concepts (entropy, information, binary Information Sources, Information Transmission Rate), and fluctuation measures (Standard Deviation and Root Mean Square). In Section 3, I analyzed the quotients $\frac{ITR}{\sigma }$ and $\frac{ITR}{V}$. Section 4 contains the discussion and final conclusions.

## 2. Theoretical Background and Methods

To introduce the necessary notation, I briefly recall Shannon Information Theory’s basic concepts [9,11,12], i.e., Information, Entropy, Information Source, and Information Transmission Rate.

### 2.1. Shannon’s Entropy and Information Transmission Rate

Let $Z^{L}$ be a set of all words of length *L*, built of symbols (letters) from some finite alphabet *Z*. Each word $w\in Z^{L}$ can be treated as an encoded message sent by Information Source Z, being a stationary stochastic process. If P(w) denotes the probability that the word $w\in Z^{L}$ already occurs, then the information in the Shannon sense carried by this word is defined as

(1) $$I\left(w\right):=-\log_{2}P(w).$$

This means that less probable events carry more information. Thus, the average information of the random variable $Z^{L}$ associated with the words of length *L* is called the Shannon block entropy and is given by

(2) $$H\left(Z^{L}\right):=-\sum_{w\in Z^{L}}P\left(w\right)\log_{2}P\left(w\right).$$

The appropriate measure for estimation of transmission efficiency of an Information Source Z is the information transmitted on average by a single symbol, i.e., ITR [9,12]

(3) $$ITR^{\left(L\right)}\left(Z\right):=\frac{1}{L}H\left(Z^{L}\right)$$

(4) $$ITR\left(Z\right)=\lim_{L\to \infty }\frac{1}{L}H\left(Z^{L}\right).$$

This limit exists if and only if the stochastic process Z is stationary [9].

In the special case of a two-letter alphabet Z={0,1} and the length of words L=1, I introduce the following notation

(5) $$H_{2}\left(p\right):=H\left(Z^{1}\right)=-p\log_{2}p-\left(1-p\right)\log_{2}\left(1-p\right).$$

where P(1)=p,P(0)=1−p are associated probabilities. This is, in fact, the formula for the entropy rate of a Bernoulli source [12]. Index 2 in (5) indicates that I consider logarithm with base 2, meaning that I consider the information expressed in bits.

### 2.2. Information Sources

In general, Information Sources are modeled as stationary stochastic processes [9,12]. The information is represented by trajectories of such processes. Here, to study the relation between Information Transmission Rate (ITR) and trajectories fluctuations, I consider Information Sources which are modeled as two-states Markov processes. The trajectories of these processes can be treated as encoded spike-trains [3,10,43]. The commonly accepted natural encoding procedure leads to binary sequences [10,43]. Spike-trains are, in fact, the main objects that carry information [3,7]. I additionally consider among the Markov processes, as a special case, the Bernoulli processes.

#### 2.2.1. Information Sources—Markov Processes

I consider a time-discrete, two-states Markov process M, which is defined by a set of conditional probabilities $p_{j|i}$, which describe the transition from state *i* to state *j*, where i,j=0,1, and by the initial probabilities $P_{0}\left(0\right),P_{0}\left(1\right)$. The Markov transition probability matrix P can be written as

(6) $$P:=\left[\begin{array}{cc} p_{0|0} & p_{0|1}\\ p_{1|0} & p_{1|1} \end{array}\right]=\left[\begin{array}{cc} 1-p_{1|0} & p_{0|1}\\ p_{1|0} & 1-p_{0|1} \end{array}\right].$$

Each of the columns of the transition probability matrix P has to sum to 1 (i.e., it is a stochastic matrix [9]).

The time evolution of the states probabilities is governed by the Master Equation [34]

(7) $$\left[\begin{array}{c} P_{n+1}\left(0\right)\\ P_{n+1}\left(1\right) \end{array}\right]=\left[\begin{array}{cc} 1-p_{1|0} & p_{0|1}\\ p_{1|0} & 1-p_{0|1} \end{array}\right]\cdot \left[\begin{array}{c} P_{n}\left(0\right)\\ P_{n}\left(1\right) \end{array}\right]$$

where *n* stands for time, $P_{n}\left(0\right),P_{n}\left(1\right)$ are probabilities of finding states “0” and “1” at time *n*, respectively. The stationary solution of (7) is given by

(8) $$\left[\begin{array}{c} P_{eq}\left(0\right)\\ P_{eq}\left(1\right) \end{array}\right]=\left[\begin{array}{c} \frac{p_{0|1}}{\left(p_{0|1}+p_{1|0}\right)}\\ \frac{p_{1|0}}{\left(p_{0|1}+p_{1|0}\right)} \end{array}\right].$$

It is known [9,12] that for Markov process M, the Information Transmission Rate as defined by (4) is of the following form

(9) $${ITR}^{M}=P_{eq}\left(0\right)\cdot H\left(p_{1|0}\right)+P_{eq}\left(1\right)\cdot H\left(p_{0|1}\right)$$

In previous papers [40,41,42], when I studied the relation between ITRs and firing rates, and when I compared ITR for Markov processes and for corresponding Bernoulli processes, I introduced a parameter *s*, which can be interpreted as the tendency of a transition from the no-spike state (“0”) to the spike state (“1”) and *vice versa*:

(10) $$s:=p_{0|1}+p_{1|0}$$

It turned out that this parameter plays an essential role in our considerations in this paper also. Note that s=2−trP and 0≤s≤2. One can observe that two-states Markov processes are Bernoulli processes if and only if s=1.

#### 2.2.2. Information Sources—Bernoulli Process Case

The Bernoulli processes play a special role among the Markov processes. Bernoulli process is a stochastic stationary process Z$=(Z_{i}),i=1,2,\ldots$ formed by binary identically distributed and independent random variables $Z_{i}$. In the case of the encoded spike-trains, I assume that the corresponding process (to be more precise, its trajectories) takes successively the values 1 (when spike has arrived in the bin) or 0 (when spike has not arrived). I assume that for a given size of time-bin applied (this depends, in turn, on the time resolution assumed), spike trains are encoded [44] in such a way that 1 is generated with probability *p*, and 0 is generated with probability *q*, where *q* is equal to 1−p. Following the definition, the Information Transmission Rate (3) of the Bernoulli process is

(11) $${ITR}^{B}\left(p,q\right)=-p\log_{2}p-q\log q=H_{2}\left(p\right).$$

#### 2.2.3. Generalized Entropy Variants

**Figure 1:**
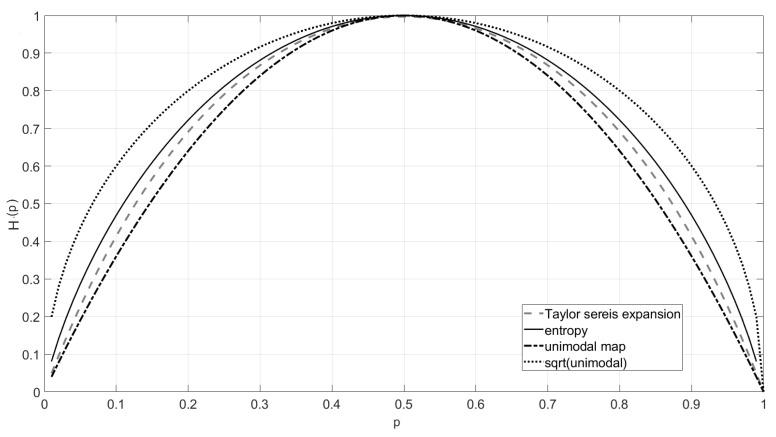
Approximation of the Shannon entropy (black solid lines) using the Taylor series expression (gray dash-dot line, 10 first terms), unimodal function (black dash line), and unimodal map root (black point line).

The form of entropy *H* was derived under assumptions of monotonicity, joint entropy, continuity properties, and Grouping Axiom. In the classical case of the entropy rate $H^{M}$ for the Markov process, in Formula (11), the terms $H(p_{1|0})$ and $H(p_{0|1})$ are clearly understood in the Shannon sense (2). To ontain a better insight into the asymptotic behavior of the relations studied in this paper, I additionally consider Formula (11) with *H* replaced by its Taylor approximation (10 terms). I also studied the interesting case when instead of *H*, I used a well known unimodal map U(p)=4p(1−p) [45] which is, in fact, close (Figure 1) to *H* in the supremum norm [46]. This idea is along the research direction related to generalized concepts of entropy developed, starting from Renyai [47], by many authors [48,49,50,51,52]. Figure 1 shows the approximation of entropy (11) by polynomials: unimodal map (black dash line) and 10 first terms in the Taylor series of *H* (gray dash-dot line). I also included the square root of the unimodal map (black point line) in this figure.

### 2.3. Fluctuations Measure

It is commonly accepted that for a given random variable X, the fluctuations of values of this random variable around its average can be characterized by the Standard Deviation σ [35]

(12) $$\sigma :={\left(E{\left(X-EX\right)}^{2}\right)}^{\frac{1}{2}}$$

where symbol E means the average taken over the probability distribution associated with the values reached by X.

Considering a stochastic process Y$\;=(X_{k}),k=1,2,3,\ldots$, where $X_{k}$ are random variables each with the same probability distribution as X, the fluctuation of trajectories of this process can be estimated by the Root-Mean-Square (RMS). For a given trajectory ${\left(x_{k}\right)}_{k=1}^{n},k=1,\ldots ,n$ RMS is defined as the root from the arithmetic mean value of the squares, i.e.,

(13) $$RMS\left(Y\right):={\left(\frac{1}{n}\sum_{k=1}^{n}{\left(x_{k}-x_{n_{avr}}\right)}^{2}\right)}^{\frac{1}{2}}$$

where $x_{n_{avr}}$ is the average value, i.e., $x_{n_{avr}}=\frac{1}{n}\sum_{k=1}^{n}x_{k}$. Note, that from this formula, the form of σ for Markov processes can be derived when using stationary distribution (8) in Formula (12).

The Standard Deviation σ for any random variable depends, in fact, not only on its probability distribution, but also on the values taken by this random variable. Here, I am interested in bits oscillation, i.e., if the spike train occurs or not. Thus, I have limited our considerations to the values 0 and 1.

To gain a better insight into the relation between ITR and signal/bits fluctuations, I also included an analysis of the quotient $\frac{ITR}{V}$. This is interesting due to the specific form of Variation for the Bernoulli process, which leads to interesting observations when one considers, for example, the unimodal map to approximate entropy (5). Moreover, when studying $\frac{ITR}{V}$ I, in fact, refer the quotient $\frac{ITR}{\sigma }$ to σ since I have simply $\frac{\left(\frac{ITR}{\sigma }\right)}{\sigma }$=$\frac{ITR}{V}$.

## 3. Results

In this section, I study the quotients $\frac{ITR}{\sigma }$ and $\frac{ITR}{V}$ as a function of the transition probability $p_{1|0}$ from the state no-spike “0” to the spike state “1” for a fixed parameter *s* (10). Note, that the probability $0<p_{1|0}<1$ and parameter 0<s<2 uniquely determined the transition probability matrix P (6) and consequently, they completely define the Markov process M, provided that initial probabilities $P_{0}\left(0\right),P_{0}\left(1\right)$ are chosen. Here, as initial probabilities, to get a stationary process, I must assume the probabilities of the form (8). To study the relation of ITR and σ, I decided to consider the quotients of these two quantities, which seems to be natural and already easy to interpret. This idea was successfully applied in [40,41,42], which compared ITR and signal correlations, as well as the frequency of jumps. I found that the key role is played by the parameter *s*, the value of which determines the qualitative and quantitative form of these relations. In fact, ITR and σ depend on *s*, and this is the reason why I analyze the quotient for fixed *s*.

### 3.1. Information against Fluctuations for Two-States Markov Processes—General Case

I start my considerations from the most general form of the two-states Markov process. To analyze the quotients $\frac{ITR}{\sigma }$ and $\frac{ITR}{V}$, I first express Standard Deviation of Markov process M in terms of conditional probability $p_{1|0}$ and parameter *s*.

#### 3.1.1. Standard Deviation in the Markov Process Case

For a given Markov process M to evaluate its fluctuation, specifically to address its long time behavior, one considers its corresponding stationary probabilities as defined by (8). Thus, in the limiting case, the Standard Deviation σ for the Markov process can be assumed as

(14) $$\sigma^{M}=\sqrt{P_{eq}\left(0\right)\cdot P_{eq}\left(1\right)}.$$

Fixing parameter *s* and expressing $\sigma^{M}$ as a function of the conditional probability $p_{1|0}$, I came to the following formula:

(15) $$\sigma_{s}^{M}\left(p_{1|0}\right)=\sqrt{\frac{p_{0|1}}{s}\cdot \frac{p_{1|0}}{s}}=\frac{\sqrt{\left(s-p_{1|0}\right)p_{1|0}}}{s}.$$

Note that in the case of Variance $\left[V_{s}^{M}\left(p_{1|0}\right)\right]={\left[\sigma_{s}^{M}\left(p_{1|0}\right)\right]}^{2}$, I have a polynomial dependence on $p_{1|0}$ (keeping in mind that *s* is fixed).

#### 3.1.2. Relation between Information Transmission Rate ITR of Markov Process and Its Standard Deviation

Let us start by establishing the relation between Standard Deviation and ITR for the Bernoulli process. This means that in our notation, *s* is equal to 1. Making use of the classical inequality x−1≥lnx (for all x>0) and doing a few simple operations, one can come to the inequality $2\cdot \log_{2}e\leq \frac{ITR_{2}\left(p_{1|0}\right)}{\sigma^{2}}$. To find the relations between entropy ${ITR}^{M}$ and $\sigma^{M}$ in more general cases, one can consider the quotient

(16) $$Q_{\sigma }^{M,s}\left(p_{1|0}\right):=\frac{{ITR}^{M,s}\left(p_{1|0}\right)}{\sigma_{s}^{M}\left(p_{1|0}\right)}.$$

Note that $Q_{\sigma }^{M,s}\left(p_{1|0}\right)$ is a symmetric function with respect to to the axe $p_{1|0}=\frac{s}{2}$, i.e.,

(17) $$Q_{\sigma }^{M,s}\left(p_{1|0}\right)=Q_{\sigma }^{M,s}\left(s-p_{1|0}\right).$$

For 0≤s≤2, I consider the quotient $Q_{\sigma }^{M,s}\left(p_{1|0}\right)$ in two cases taking into account the range of $p_{1|0}$

(18) $$\left(A\right)\;0\leq s\leq 1\;\mathrm{and}\;\mathrm{this}\;\mathrm{implies}\;0\leq p_{1|0}\leq s$$

(19) $$\left(B\right)\;1<s<2\;\mathrm{and}\;\mathrm{this}\;\mathrm{implies}\;s-1\leq p_{1|0}\leq 1.$$

Substituting (8), (10) and (14) into (16) I obtain

(20) $$Q_{\sigma }^{M,s}\left(p_{1|0}\right):=\frac{\frac{p_{0|1}}{s}H\left(p_{1|0}\right)+\frac{p_{1|0}}{s}H\left(p_{0|1}\right)}{\frac{\sqrt{\left(s-p_{1|0}\right)p_{1|0}}}{s}}$$

and after simple calculations, I have

(21) $$\begin{array}{c} Q_{\sigma }^{M,s}\left(p_{1|0}\right)=\frac{\frac{s-p_{1|0}}{s}H\left(p_{1|0}\right)+\frac{p_{1|0}}{s}H\left(s-p_{1|0}\right)}{\sqrt{\frac{s-p_{1|0}}{s}\cdot \frac{p_{1|0}}{s}}}\\ =\sqrt{\frac{s-p_{1|0}}{p_{1|0}}}H\left(p_{1|0}\right)+\sqrt{\frac{p_{1|0}}{s-p_{1|0}}}H\left(s-p_{1|0}\right) \end{array}$$

One can check that for smaller s∈(0,1), i.e., in case (18), for a given fixed *s* when $p_{1|0}$ tends to interval bounds 0 or to *s*, the quotient $Q_{\sigma }^{M,s}\left(p_{1|0}\right)$ tends to 0, i.e.,

(22) $$\lim_{p_{1|0}\to 0^{+}}Q_{\sigma }^{M,s}\left(p_{1|0}\right)=\lim_{p_{1|0}\to s^{-}}Q_{\sigma }^{M,s}\left(p_{1|0}\right)=0.$$

By the form of (20) and symmetry property (17) it is clear that the quotient $Q_{\sigma }^{M,s}\left(p_{1|0}\right)$ reaches the maximum in the symmetry point $p_{1|0}=\frac{s}{2}$ and it is equal to

(23) $$Q_{\sigma }^{M,s}\left(\frac{s}{2}\right)=2H\left(\frac{s}{2}\right).$$

One can check that in the case (B), i.e., for s∈(1,2) for a given fixed *s* when $p_{1|0}$ tends to s−1 or to 1 the quotient $Q_{\sigma }^{M,s}\left(p_{1|0}\right)$ tends to $\frac{H(s-1)}{\sqrt{\left(s-1\right)}}$, i.e.,

(24) $$\lim_{p_{1|0}\to {\left(s-1\right)}^{+}}Q_{\sigma }^{M,s}\left(p_{1|0}\right)=\lim_{p_{1|0}\to 1^{-}}Q_{\sigma }^{M,s}\left(p_{1|0}\right)=\frac{H(s-1)}{\sqrt{s-1}}.$$

Thus, I have for s∈(1,2)

(25) $$\frac{H(s-1)}{\sqrt{\left(s-1\right)}}\leq Q_{\sigma }^{M,s}\left(p_{1|0}\right)\leq 2H\left(\frac{s}{2}\right).$$

Finally, I obtained an interesting estimation of Information Transmission Rate ITR by the level of fluctuation σ:

(26) $$\frac{H(s-1)}{\sqrt{s-1}}\sigma_{s}^{M}\left(p_{1|0}\right)\leq ITR^{M,s}\left(p_{1|0}\right)\leq 2H\left(\frac{s}{2}\right)\sigma_{s}^{M}\left(p_{1|0}\right).$$

**Figure 2:**
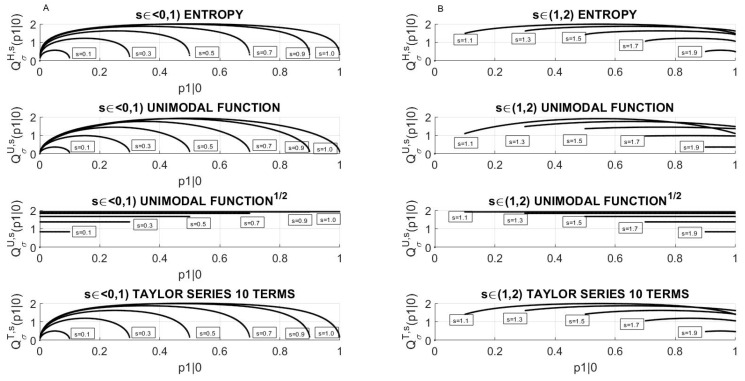
The quotient $\frac{ITR}{\sigma }$ as a function of the transition probability $p_{1|0}$ for chosen values of the jumping parameter *s*: (**A**) For parameters 0≤s≤1 due to (16) the range of $p_{1|0}$ is [0,s] and (**B**) for 1<s<2 according to (17) the range of $p_{1|0}$ is $s-1\leq p_{1|0}\leq 1$. The courses of the quotients $Q_{\sigma }^{H,s}\left(p_{1|0}\right),Q_{\sigma }^{U,s}\left(p_{1|0}\right),Q_{\sigma }^{T,s}\left(p_{1|0}\right)$ for Shannon form, unimodal map, unimodal map root, Taylor series being applied as *H* in Formula (9) are presented.

The typical runnings of $Q_{\sigma }^{M,s}\left(p_{1|0}\right)$ for some values of the parameter, *s* are shown in Figure 2. Column A is devoted to lower values of the jumping parameter 0≤s≤1, while column B presents the $Q_{\sigma }^{M,s}$ courses for higher values of the jumping parameter 1<s<2. Observe, that for 1<s<2, the curves intersect contrary to the case 0≤s≤1. This is mostly since the limiting value (24) is not a monotonic function of *s*, while the maximal value (23) is already monotonic.

Note, that for the approximation of entropy *H* by polynomials, specifically by unimodal map *U* and by Taylor series *T*, the corresponding quotients $Q_{\sigma }^{U,s}$ B, $Q_{\sigma }^{T,s}$ behave similarly as for the Shannon form of *H* (see Figure 2).

#### 3.1.3. Relation between Information Transmission Rate ITR of Markov Process and Its Variation

To find how the Variation of trajectories of Markov Information Source affects the Information Transmission Rate, one should consider a modified quotient

(27) $$Q_{\sigma }^{M,s}\left(p_{1|0}\right)=\frac{{ITR}^{M}\left(p_{1|0}\right)}{V(p_{1|0})}=\frac{{ITR}^{M}\left(p_{1|0}\right)}{P_{eq}\left(0\right)\cdot P_{eq}\left(1\right)}.$$

Substituting (8) and (10) to (27) I obtain

(28) $$Q_{V}^{M,s}\left(p_{1|0}\right)=\frac{\frac{p_{0|1}}{s}H\left(p_{1|0}\right)+\frac{p_{1|0}}{s}H\left(p_{0|1}\right)}{\frac{p_{0|1}}{s}\cdot \frac{p_{1|0}}{s}}=s\left[\frac{H(p_{1|0})}{p_{1|0}}+\frac{H(s-p_{1|0})}{s-p_{1|0}}\right].$$

First, observe that as in the standard deviation case, I have a symmetry property around the value $\frac{s}{2}$, i.e.,

(29) $$Q_{V}^{M,s}\left(p_{1|0}\right)=Q_{V}^{M,s}\left(s-p_{1|0}\right).$$

By this symmetry, it is clear that $Q_{V}^{M,s}\left(p_{1|0}\right)$ reaches extremum at the point $p_{1|0}=\frac{s}{2}$ and it is equal to $4H(\frac{s}{2})$.

Observe, that in the case (A), i.e., for a given fixed s∈(0,1), for $p_{1|0}$ tending interval bound, i.e., to 0 or s− the quotient $Q_{V}^{M,s}\left(p_{1|0}\right)$, in opposite to $Q_{\sigma }^{T,s}\left(p_{1|0}\right)$, tends to infinity, i.e.,

(30) $$\lim_{p_{1|0}\to 0^{+}}Q_{V}^{M,s}\left(p_{1|0}\right)=\lim_{p_{1|0}\to s^{-}}Q_{V}^{M,s}\left(p_{1|0}\right)=+\infty .$$

Thus, it is clear that $Q_{V}^{M,s}\left(p_{1|0}\right)$ reaches a minimum at the point $p_{1|0}=\frac{s}{2}$.

In the case of (B), it turned out that the quotient $Q_{V}^{M,s}\left(p_{1|0}\right)$ for any fixed s∈(1,2) is bounded both from below and from above. I have:

(31) $$\lim_{p_{1|0}\to {\left(s-1\right)}^{+}}Q_{V}^{M,s}\left(p_{1|0}\right)=\lim_{p_{1|0}\to 1^{-}}Q_{V}^{M,s}\left(p_{1|0}\right)=s\frac{H(s-1)}{s-1}.$$

Numerical calculations showed that for the parameters $s>s_{0}$ the point $p_{1|0}=\frac{s}{2}$ is a minimum while for $s<s_{0}$ at this point, there is a maximum, where the critical parameter $s_{0}\approx$1.33 can be calculated from the equality:

(32) $$s_{0}\frac{H(s_{0}-1)}{s_{0}-1}=4H\left(\frac{s_{0}}{2}\right).$$

**Figure 3:**
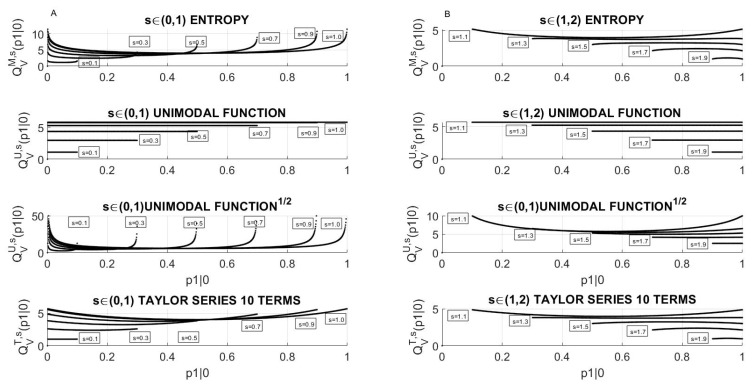
The quotient $\frac{ITR}{V}$ as a function of the initial probability $p_{1|0}$ for the chosen values of the jumping parameter *s*: (**A**) For parameters 0≤s≤1 due to (16) the range is $0<p_{1|0}<s$ and (**B**) For parameters 1<s<2 due to (16) the range is $s-1\leq p_{1|0}\leq 1$. Observe that $\frac{ITR}{V}$ has a completely different course to that of the quotient $\frac{ITR}{\sigma }$ presented in Figure 2.

The typical running of the $Q_{V}^{M,s}\left(p_{1|0}\right)$ for some values of the parameter, *s* is shown in Figure 3. Panel A (left column) is devoted to lower values of the jumping parameter 0≤s≤1, while panel B presents graphs of $Q_{V}^{M,s}\left(p_{1|0}\right)$ for higher values of the jumping parameter 1<s<2.

It turned out that the approximation of entropy *H* by polynomials, namely by the unimodal map and by Taylor series, leads to completely different behavior of $Q_{V}^{M,s}\left(p_{1|0}\right)$. Note, that for the approximation of *H* in (2) with the unimodal map the quotient $Q_{V}^{U,s}\left(p_{1|0}\right)$, for each *s*, is a constant and equal to 4s(2−s), while for the approximation by the Taylor series (10 terms), the quotient $Q_{V}^{T,s}\left(p_{1|0}\right)$ preserves a similar course as thay of *H* of the Shannon form.

## 4. Discussion and Conclusions

In this paper, I studied the relation between the Information Transmission Rate carried out by sequences of bits and the fluctuations of these bits. These sequences originate from Information Sources which are modeled by Markov processes. During the last 30 years, authors have modeled neuron activity by different variants of Markov processes, e.g., inhomogeneous Markov Interval Models and Hidden Markov Processes [20,21,22]. The Poisson Point processes commonly used to model experimental data of neuronal activity also exhibit the Markov property [18,19]. Our results show that the qualitative and quantitative character of the relation between the Information Transmission Rate and fluctuations of signal bits strongly depends on the jumping parameter *s*, which we introduced in our previous papers [41,42]. This parameter characterizes the tendency of the process to transition from state to state. In some sense, it describes the variability of the signals.

It turned out that, similarly as in our previous papers, when have studied the relation between Information Transmission Rates, spikes correlations, and frequencies of these spikes appearance, the critical value of *s* was equal to 1, which corresponds to the Bernoulli process. For all small *s*(s<1), the quotient $\frac{ITR}{\sigma }$ could reach 0, while for larger *s*(s>1), this quotient was always separated from 0. Specifically, for 1<s<1.7, the ITR will always be, independent of transition probabilities which form this *s*, above the level of fluctuations (i.e., σ<ITR). Thus, this highlights an interesting fact that for large enough *s*, the information is never completely lost, independent of the level of fluctuations.

On the other hand, for each 0<s<2, the quotient $\frac{ITR}{\sigma }$ is limited from above by 2 and it is reached for each *s*, for $p_{1|0}=\frac{s}{2}$ Thus, I have that the maximum is reached when $p_{1|0}=p_{0|1}$. This means that, when one compares ITR to σ, the most effective transmission was for symmetric communication channels. Note, that the capacity C(s) of such channels is already equal to

(33) $$C\left(s\right)=1-H\left(\frac{s}{2}\right).$$

Additionally, it turned out that $\frac{ITR}{\sigma }$ for the approximation of Shannon entropy *H* by polynomials, specifically by the unimodal map and its Taylor series, behaves similarly. Observe, that for all *s* these quotients, independent of the approximation applied, reach the maximum for $p_{1|0}$ equal to $\frac{s}{2}$ and monotonically increase for $p_{1|0}$ less than $\frac{s}{2}$, while monotonically decrease for $p_{1|0}$ below $\frac{s}{2}$.

For a better insight into the relation between ITR and signal variability, I also referred ITR to Variance. I observed that the behavior of the $\frac{ITR}{V}$ significantly differs from the behavior of $\frac{ITR}{\sigma }$. For each s<1, the quotient $\frac{ITR}{V}$ can tend to infinity and it is separated from 0. For 1<s<2, it is limited from above and it never reaches 0 for any *s*. However, it behaves in a more complex way than $\frac{ITR}{\sigma }$ by having even three local extreme points, e.g., it is visible for s=1.3 and s=1.5. On the other hand, approximations of Shannon entropy *H* by polynomials such as the unimodal map or by its Taylor series, contrary to the case of $\frac{ITR}{\sigma }$, lead to a significant qualitative difference between the behavior of $\frac{ITR}{V}$.

To summarize, the results obtained show that for Markov information sources, regardless of the level of fluctuation, the level of Information Transmission Rate does not reduce to zero, provided that the transition parameter s is sufficiently large. This means that to obtain more reliable communication, the spike trains should have a higher tendency of transition from the state no spike to spike state and vice versa. The inequality (26) allows for estimatio of the amount of information being transmitted by the level of signal fluctuations. Signal fluctuations characterize, in fact, the level of noise.

The results are presented in the context of signal processing in the brain, due to the fact that information transmission in the brain is in this case a natural and fundamental phenomena. However, our results have, in fact, a general character and can be applied to any communication system modeled by two-states Markov processes.
